# Labor Supply and Well-Being During the Early Stages of the COVID-19 Crisis in the Netherlands: Lessons from Microdata

**DOI:** 10.1007/978-3-030-65355-2_10

**Published:** 2021-03-20

**Authors:** Hans-Martin von Gaudecker, Bettina Siflinger

**Affiliations:** 1 Tilburg University, Tilburg, The Netherlands; 2 Tilburg University, Tilburg, The Netherlands; 3 Tilburg University, Tilburg, The Netherlands; 4 Tilburg University, Tilburg, The Netherlands; 5 Institute for Applied Microeconomics, University of Bonn, Bonn, Germany; 6 Department of Econometrics and Operations Research, Tilburg School of Economics and Management, Tilburg, The Netherlands

## Abstract

Like many other countries, the Netherlands shut down large parts of economic and social life in the spring of 2020 in response to the COVID-19 pandemic. Between late March and early May, schools and childcare facilities as well as restaurants, cafes, and bars were shut down; contact-related occupations were closed; gatherings were prohibited; and employees were advised to work from home as much as possible. While these regulations represented a sharp cut in individuals’ personal lives, they were more relaxed in the Netherlands than in many other European countries. At the same time, the Netherlands has enacted large-scale economic relief programs.

This chapter gives an overview of how labor supply and well-being have changed in the Netherlands in the early stage of the COVID-19 pandemic. We show that changes in the labor market have affected different groups of people differently and we discuss reasons for these differences. In addition, we illustrate how the consequences of the lockdown have altered the well-being of Dutch workers.

Like many other countries, the Netherlands shut down large parts of economic and social life in the spring of 2020 in response to the COVID-19 pandemic. Between late March and early May, schools and childcare facilities as well as restaurants, cafes, and bars were shut down; contact-related occupations were closed; gatherings were prohibited; and employees were advised to work from home as much as possible. While these regulations represented a sharp cut in individuals’ personal lives they were more relaxed in the Netherlands than in many other European countries.^1^ At the same time, the Netherlands has enacted large-scale economic relief programs.

This chapter gives an overview of how labor supply and well-being have changed in the Netherlands in the early stage of the COVID-19 pandemic. We show that changes in the labor market have affected different groups of people differently and we discuss reasons for these differences. In addition, we illustrate how the consequences of the lockdown have altered the well-being of Dutch workers.

## Data: The LISS Panel

To investigate the consequences of the COVID-19 crisis for the Dutch population, we designed and fielded several questionnaires asking members of the Longitudinal Internet Studies for the Social Sciences (LISS) panel about behaviors, beliefs, and expectations during the COVID-19 crisis. The first module was fielded between March 20 and March 31, 2020, a few days into the lockdown. Three modules followed in April, May, and June. In addition, we collected time use and consumption data to analyze how individuals had allocated time and money during the lockdown. The LISS panel is based on a probability sample of individuals registered by Statistics Netherlands; it has been running since 2007 and constitutes roughly 4000 Dutch households comprising about 7000 individuals. It is administered by CentERdata, a survey research institute affiliated with Tilburg University. For all modules fielded so far, response rates for our questionnaire were in excess of 80%, which translates into a longitudinal sample of about 5000 individuals.

## The Number of Working Hours

All over the world, social distancing measures led to an immediate increase in unemployment rates and a decrease in working hours. Studies analyzing samples of workers in the United States and the United Kingdom show that about 60% of respondents claim to have worked fewer hours. The share of workers who lost their employment (probably or definitely) due to the virus was 12% (US) and 9% (UK) (see e.g., Adams-Prassl et al. 2020b; Coibion et al. 2020; Bick and Blandin 2020). In the Netherlands, these numbers are much lower: about 27% of the respondents reported to work fewer hours, and about 3% of the respondents worked zero hours in late March 2020. While overall these numbers suggest a less severe early impact of the pandemic on labor supply, we find remarkable differences by education.

**Fig. 10.1:**
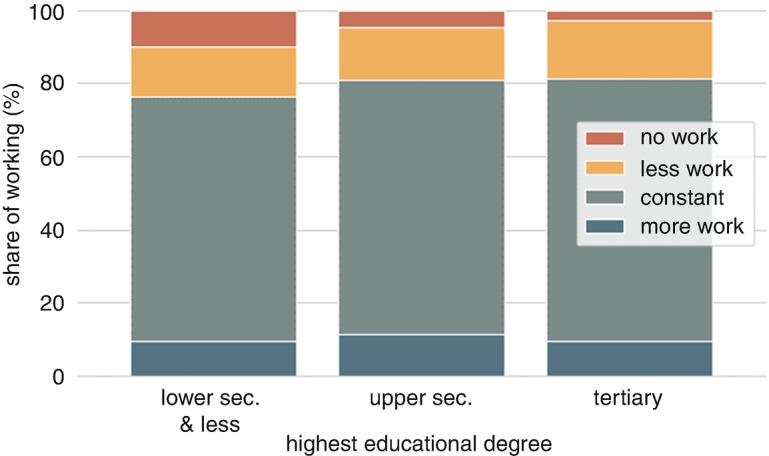
Share of workers with different changes in the number of working hours, by the level of education (data source: LISS)

Figure 10.1 shows that the least-educated group in our sample is most likely to work zero hours. Almost 10% of the low-educated work zero hours, which is twice as high as in the middle education group and almost four times higher than among those with a tertiary degree. By contrast, the share of workers with reduced hours is at around 13.5% for the least educated and rises in education. Working more hours is more prevalent among those with upper secondary education (a bit more than 11%) than in the other groups (von Gaudecker et al. 2020).

**Fig. 10.2:**
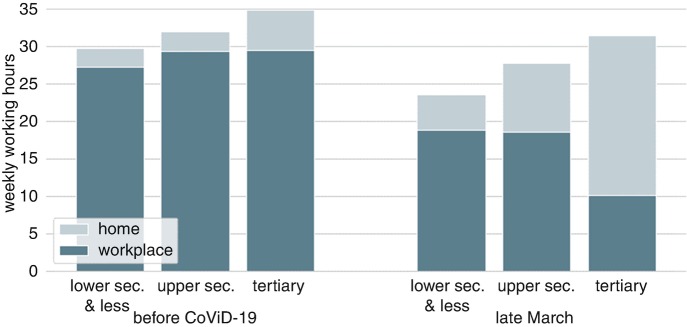
Working hours at the workplace and at home, by the level of education (data source: LISS)

Figure 10.2 breaks down the change in total working hours by those performed at the usual workplace and at home by education. The COVID-19 pandemic strongly amplifies pre-crisis differences in shares of hours worked from home and the total weekly working hours between low, medium, and highly educated individuals. This pattern intensifies differences in the total hours worked among education groups. While both low- and medium-educated individuals worked a bit more than 8% of their hours from home before the COVID-19 pandemic, the highly educated had a share in excess of 15%. During the onset of the COVID-19 crisis, all groups increased their home office shares. However, hours worked from home almost quadrupled among the highly educated (+16 h), while they tripled for the medium educated (+6.5 h) and not even doubled for the low educated (+2.2 h).

Investigating the reduction in working hours reveals a similar divide. Pre-crisis working hours are lowest among low educated, and highest among highly educated, with the medium educated somewhere in between (low: 29.8, medium: 32, high: 34.9 weekly hours). The onset of the COVID-19 pandemic strongly magnifies this division. Low-educated workers experience the largest decrease in terms of absolute and relative working hours (–21%), medium-educated workers the second-largest decrease (–13%), while the decrease is less than 10% for highly educated workers. A logical explanation for the stronger reduction in total working hours for lower-educated individuals is that their jobs are associated with a lower flexibility to work from home. Three mechanisms come to mind. First, differences in the task compositions of the respective jobs can explain why some jobs can be done from home more easily compared to others. Second, if set-up costs are involved (e.g., laptop by the employer) and/or facilities to work from home are limited (VPN connections); employers might be forced to allow only a part of the workforce to work from home. Third, the share of essential workers (“essentiele beroepen en cruciale sectoren”) rises in education (von Gaudecker et al. 2020).

## The Differences Between Men and Women

One question that naturally arises in the analysis of labor supply is whether men and women react in different ways. When analyzing women’s labor supply, we find that their total working hours dropped from 30.4 to 25.5 (–17%) since the beginning of the crisis. Men worked 38.8 h on average before COVID-19 and 34.8 h afterwards (–10%). The gender difference in the decrease in hours worked is 0.87 h in absolute terms and 7% in relative terms. An additional analysis reveals that women are affected more strongly by the pandemic in both extremes: 13% worked longer hours in late March, but, at the same time, 22% reported a reduction in their working hours or do not work at all. For men, these numbers are 7% and 17%, respectively. Put differently, less than two-thirds of the women but more than three-quarters of the men worked the same hours in late March as they did before. While very large reductions in absolute loss of hours occur mainly for men, a larger share of women faced smaller reductions.

We propose two potential explanations for the observed changes in female working hours. First, women disproportionally work in sectors and occupations that are considered “essential” and this thus raises female working hours. Our data indicate that 20% of the men and 34% of the women work in essential occupations. These are mostly concentrated in the health care and welfare sector and in education, sectors in which women make up a particularly large share of the workers (82% in health care and welfare, 63% in education). Second, mothers may work less in total during the lockdown because they have childcare responsibilities. However, our time use data do not show that this latter explanation concerns large numbers in quantitative terms—we do not find meaningful differences in total hours worked between mothers of young children and other women. However, the share of home office work does react to the presence of young children for both genders.

## Well-Being and Mental Health

Combining childcare and work is stressful. Together with the extraordinary amount of economic uncertainty, this could be a perfect storm for impairing parents’ well-being and mental health. We thus turn to showing the development of mental health between November 2019 and May 2020 among the working population, zooming in on working parents with young children below. Mental health is measured using the five-item mental health inventory (MHI-5), a validated instrument for assessing mental health in adults. It ranges from 0 to 100, with higher values representing better mental health. Respondents with a value of below 60 are considered to be at risk for mental health problems. We use this cut-off to illustrate the development of mental health problems in November 2019, late March 2020, and May 2020.

**Fig. 10.3:**
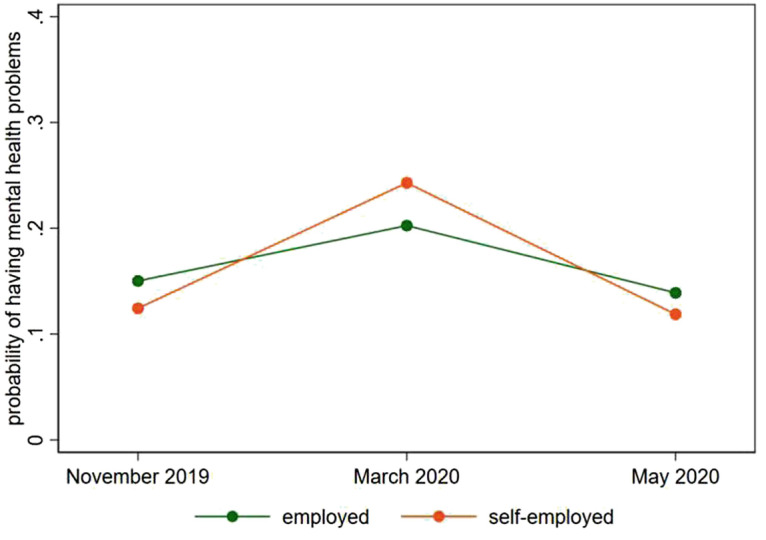
Mental health problems before and during the lockdown, employed and self-employed respondents (data source: LISS)

Figure 10.3 shows the development of mental health problems for employed and self-employed respondents. In November 2019, 12.4% of the self-employed and 15% of the employees experienced mental health problems. Shortly after the lockdown (March 2020), the share of self-employed people with mental health problems almost doubled (24.3%), exceeding the fraction of employed people with mental health problems (20.3%). After about 2 months into the lockdown, mental health problems reverted to pre-crisis levels for both groups of workers. The following picture is drawn in Fig. 10.3.

While the COVID-19 crisis suddenly increased economic uncertainty and stress for the working population, workers learned about their situation over the course of the crisis, and the governmental programs announced took effect. This probably led to a reduction in stress and uncertainty, such that the mental health problems decreased to pre-crisis levels in the working population overall.

**Fig. 10.4:**
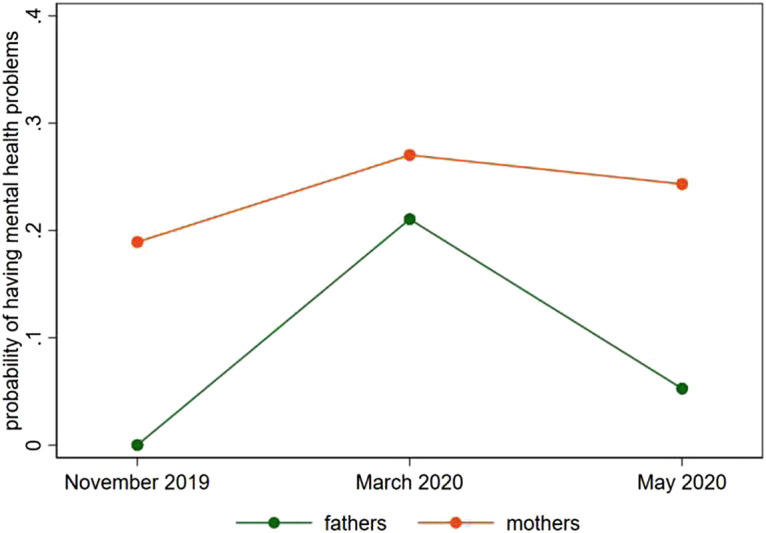
Mental health problems before and during the lockdown, working parents with young children (data source: LISS)

As mentioned before, the closure of schools and childcare facilities forced parents to find alternative care arrangements or to take care of the children themselves while working from home. This double burden may have created an enormous amount of stress for working parents. Figure 10.4 shows the share of mental health problems among mothers and fathers who took care of their young children (below the age of 12). In November 2019, no father in our sample reported signs of mental health problems while about 19% of the mothers experienced mental health problems. These shares were significantly higher in March 2020. The share of mothers with mental health problems increased to 27%. Fathers experienced an even stronger increase in mental health problems, almost catching up with mothers (21%). Later during the crisis, mental health problems decreased for both groups. Still, the fraction of fathers and mothers with mental health problems is higher than in November 2019 (5% and 24%).

Thus, even 2 months after the lockdown was enacted parents still suffered from the double burden of working from home and taking care of their children.^2^

## Conclusion

Unlike other crises, the COVID-19 pandemic had a very sharp onset. Literally overnight, it led to dramatic shifts in the way work and childcare was organized. Using data collected at high frequency over the first weeks and months of the crisis, we have documented the huge shift towards working from home and the heterogeneity across population groups. After a huge spike in late March, the mental well-being of the overall working population reverted to pre-crisis levels in May. However, for parents with young children, mental health problems continued to be significantly more frequent than before the crisis. It will be important to track these and other developments in the months and years to come.

## Footnotes

1 Regarding these regulations, the Oxford Response Stringency Index (Hale et al. 2020) assigns the Netherlands scores between 81 and 86 for the second half of March 2020, placing it close to Germany. It is well below countries like Italy and France that enacted stricter lockdowns and above countries like the UK or the USA, which pursued less stringent policies at that point in time.

2 For the USA, Adams-Prassl et al. (2020a) find negative mental health effects of the lockdown for women only.
